# Effects of Auditory Stimuli in the Horizontal Plane on Audiovisual Integration: An Event-Related Potential Study

**DOI:** 10.1371/journal.pone.0066402

**Published:** 2013-06-17

**Authors:** Weiping Yang, Qi Li, Tatsuya Ochi, Jingjing Yang, Yulin Gao, Xiaoyu Tang, Satoshi Takahashi, Jinglong Wu

**Affiliations:** 1 Biomedical Engineering Laboratory, Graduate School of Natural Science and Technology, Okayama University, Okayama, Japan; 2 School of Computer Science and Technology, Changchun University of Science and Technology, Changchun, China; University of Salamanca- Institute for Neuroscience of Castille and Leon and Medical School, Spain

## Abstract

This article aims to investigate whether auditory stimuli in the horizontal plane, particularly originating from behind the participant, affect audiovisual integration by using behavioral and event-related potential (ERP) measurements. In this study, visual stimuli were presented directly in front of the participants, auditory stimuli were presented at one location in an equidistant horizontal plane at the front (0°, the fixation point), right (90°), back (180°), or left (270°) of the participants, and audiovisual stimuli that include both visual stimuli and auditory stimuli originating from one of the four locations were simultaneously presented. These stimuli were presented randomly with equal probability; during this time, participants were asked to attend to the visual stimulus and respond promptly only to visual target stimuli (a unimodal visual target stimulus and the visual target of the audiovisual stimulus). A significant facilitation of reaction times and hit rates was obtained following audiovisual stimulation, irrespective of whether the auditory stimuli were presented in the front or back of the participant. However, no significant interactions were found between visual stimuli and auditory stimuli from the right or left. Two main ERP components related to audiovisual integration were found: first, auditory stimuli from the front location produced an ERP reaction over the right temporal area and right occipital area at approximately 160–200 milliseconds; second, auditory stimuli from the back produced a reaction over the parietal and occipital areas at approximately 360–400 milliseconds. Our results confirmed that audiovisual integration was also elicited, even though auditory stimuli were presented behind the participant, but no integration occurred when auditory stimuli were presented in the right or left spaces, suggesting that the human brain might be particularly sensitive to information received from behind than both sides.

## Introduction

In a social environment, many objects and events are often perceived simultaneously via different sensory systems. The information received from the different modalities must be localized and integrated by several systems to produce coherent cognition in the brain. Previous studies have shown that bimodal audiovisual stimuli can be discriminated or detected more accurately and faster than unimodal auditory or visual stimuli presented alone [1], [2], [3], [4]. This facilitative effect is called “audiovisual integration”. In previous studies, discrimination tasks were used to investigate audiovisual integration using target and standard stimuli, and subjects were required to respond only to target stimuli [2], [3]. Specifically, discrimination tasks involve visual (or auditory) detection methods in which subjects are instructed to respond to visual (or auditory) target stimuli while not responding to standard stimuli [3], [5] or visual and auditory detection methods in which subjects are required to respond to both visual and auditory target stimuli [6], [7].

In behavioral studies, using either visual and auditory detection [8], [9] or one modality (i.e., visual stimuli) detection [10], some researchers have shown that the facilitative effect of bimodal stimuli on signal detectability depend on the spatial location of the stimuli and that the modalities in the same location can produce the greatest response enhancements, whereas spatially disparate modalities induce a less potent effect or no change in response [11]. Moreover, previous studies based on visual discrimination task have also confirmed that the perception of visual stimuli can be improved by simultaneous auditory stimuli occurring in close spatial location to the visual stimuli [12], [13]. Recently, some researchers investigated audiovisual integration elicited by a pair of visual and auditory stimuli presented in the frontal horizontal plane (0° from the fixation point) and right locations (up to 90°from the fixation point) [14]. Their findings indicated that the facilitative effects in localizing paired visual and auditory stimuli decrease from the central to peripheral locations and that this enhancement was no longer observed for the 90° right location. However, Stein, London, Wilkinson, and Price (1996) used a visual discrimination task to determine that the perception of peripheral visual stimuli (30° to the left or right of fixation) were unaffected by an auditory stimulus that was presented at the same location as the visual stimuli or at 45° to the right or left of the visual stimuli. When the visual stimuli were presented at the central fixation point, perception and detection of the visual stimuli were enhanced by simultaneous auditory stimuli, regardless of whether the auditory stimulus was spatially congruent or displaced 45° to the right or left of fixation [15]. Therefore, the positional relationship of modalities was an unimportant factor in determining whether multisensory interactions had occurred.

In some event-related potential (ERP) studies, audiovisual (AV) integration was investigated by comparing the ERP from a bimodal AV stimulus with the sum of the ERPs of the constituent auditory (A) and visual (V) stimuli [16], [17], [18], [19]. In these studies, the formula ERP (AV) – [ERP (A)+ERP (V)] = ERP (A × V interactions) was used to estimate cross-modal interaction or integration in the differences between the brain responses to bimodal stimuli and the algebraic sum of the unimodal responses. Moreover, the [AV–(A+V)] complex was used to determine cortical regions that were uniquely activated by bimodal stimulation. The effects of AV integration were expected to be observed as differences between the AV and the (A+V) waveforms [AV–(A+V)]. Based on this method, Giard and Peronnet (1999) reported that integrative processes can occur as latencies as early as 40 milliseconds (ms) after stimulus onset over the posterior scalp areas when visual and auditory stimuli are simultaneously presented in the central location using a discrimination task [7].

Furthermore, audiovisual integration of a spatial pair of visual and auditory stimuli was investigated using visual and auditory discrimination task with ERP recordings [20], [21]. In studies by Teder-Sälejärvi et al. (2005), a pair of auditory and visual stimuli were presented either at the same spatial location (i.e., 30° to the left or right of the central fixation point) or at opposite spatial locations (i.e., V left and A right). Their findings showed that responses were faster for bimodal stimuli than for unimodal stimuli regardless of whether the stimuli were in the same or opposite spatial locations. However, Gondan et al. (2005) found that the differential integration between the same spatial location and the opposite spatial location was observed as early as 160 ms after stimulus onset over the parietal cortex using ERPs. Therefore, studies using high-density ERP recordings have revealed important insights about when (i.e., latency) and where (i.e., topography) audiovisual integration occurs in the human brain and indicated that the effect of integration was earlier in central, as opposed to peripheral, locations. These electrophysiological findings also confirmed that one modality can be affected by another modality, even when these modalities occur from incongruent spatial locations.

In these previous studies, audiovisual integration was studied using auditory and visual stimuli in various locations; however, these modalities were presented either directly in front of the participants, or to their left or right. Some recent studies have investigated audiovisual speech perception in which the range of locations for auditory stimuli and visual stimuli are extended to include those behind the participants (180° from the point of fixation) (i.e., the entire horizontal plane) [22]. Their results showed that the facilitation effect is stronger when auditory stimuli are presented behind the participants. These results indicated that audiovisual speech perception is impervious to spatial discordances due to specialized processing of more complex speech signals [23]. Moreover, Wyk et al. (2010) investigated the cortical integration of audiovisual speech and non-speech stimuli. Their findings demonstrated the differential activation of cortical networks involved in the integration of speech and non-speech stimuli [24]. However, whether simple non-speech signals originating from behind participants can enhance the detection of visual stimuli remains unclear. Second, whether simple non-speech signals originating 90° from the left or right of participants in the same horizontal plane as the “behind” signals can affect the detection of visual stimuli also remains unclear from electrophysiological evidence.

The present study investigates the effects of auditory stimuli in the horizontal plane on audiovisual integration using high density ERP recording and analyzes the neural bases of audiovisual integration in more detail. We designed a simple visual discrimination task that included visual stimuli, auditory stimuli and audiovisual stimuli, which were randomly presented with equal probability. Visual stimuli consisted of Gabor gratings, which were presented directly in front of the participants. Auditory stimuli consisted of a 3000 Hz sinusoidal tone, which was presented at one location on an equidistant horizontal plane at the front (0°, the fixation point), right (90°), back (180°), or left (270°) of the participants. By comparing the audiovisual integration elicited by the visual stimulus presented at the front location with the auditory stimuli presented at the four horizontal plane locations, we examined whether audiovisual integration can be modulated according to the positions of the auditory stimuli in the horizontal plane, particularly originating from behind the participants.

## Materials and Methods

### Participants

Fourteen healthy volunteers (ages 21–24 years, mean age 22.5 years) participated in this study. All of the participants had normal or corrected-to-normal vision and normal hearing capabilities. The participants provided written informed consent for their participation in this study, which was previously approved by the ethics committee of Okayama University.

### Stimuli and Task

The experiment was performed in a dimly lit, sound-attenuated and electrically shielded room (laboratory room, Okayama University, Japan). Streams of unimodal visual, unimodal auditory, and bimodal audiovisual stimuli (auditory and visual components that occur simultaneously) were randomly presented.

The unimodal visual stimuli consisted of two subtypes of standard and target stimuli. The visual standard stimulus was a Gabor patch with vertical gratings (2° diameter, spatial frequency = 1.6 cycles/degree). The visual target stimulus was a Gabor patch with horizontal gratings (2° diameter, spatial frequency = 1.6 cycles/degree). These visual stimuli were presented approximately 4° below the fixation point on a 21-inch computer monitor (128 background color) positioned 70 cm in the front of the subject’s eyes. The visual stimulus was titrated for each subject so that detection required highly focused attention but could be performed at approximately 80% accuracy. This adjustment was accomplished by changing the contrast. The contrast of these ranged from 3% to 7% in units of Michelson contrast: (max–min)/(max+min), with max and min being the maximal and minimal values of the Gabor patch.

The unimodal auditory stimulus was a 3000 Hz sinusoidal tone, with a linear rise and fall time of 5 milliseconds and amplitude of 60 dB. The auditory stimulus originated from one of four loudspeakers that were hidden by a black curtain. Four loudspeakers were positioned 80 cm from the front (0°, the fixation point), right (90°), back (180°), and left (270°) of the participants, at the level of the participant’s ears. The bimodal audiovisual stimulus consisted of the simultaneous presentation of both the unimodal visual stimuli (standard or target) and the unimodal auditory stimuli (originating from one of the four locations). The target stimuli were presented at a frequency of approximately 19% of the total stimuli. The duration of each type of stimulus was 40 ms. The inter-stimulus interval (ISI) varied randomly between 800 ms and 1200 ms (mean ISI = 1000 ms).

**Figure 1:**
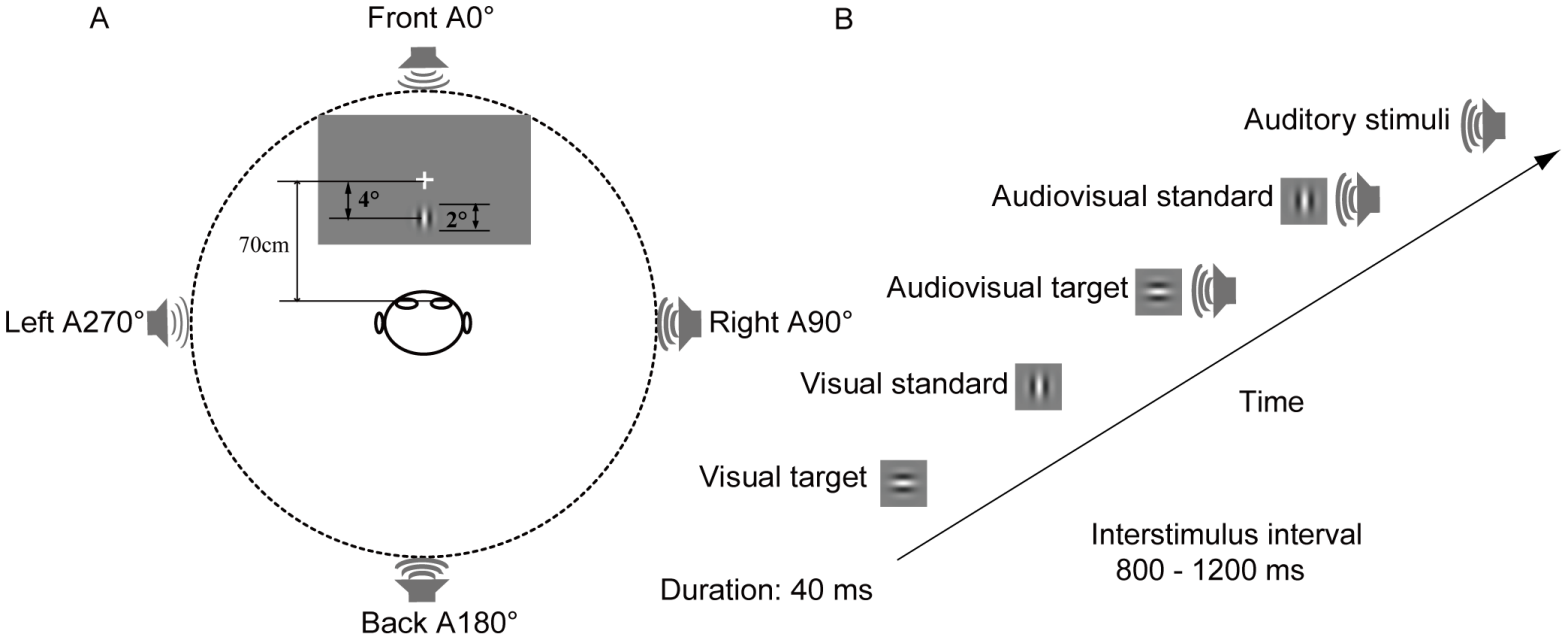
Experimental paradigm and stimuli. (A) Subjects sat approximately 70 cm from the screen. Visual stimuli were presented on the front screen. Auditory stimuli were randomly presented at one of four locations using speakers (front 0°, right 90°, back 180°, and left 270°). (B) Stimuli types.

Eight experimental blocks were performed in this study, with each block lasting approximately 4 min. Each block consisted of 266 trials in total (i.e., visual (24+10), auditory (24 × 4) and audiovisual (24 × 4+10 × 4)), in which the unimodal visual stimuli contained 24 standard stimuli and 10 target stimuli, unimodal auditory stimuli included 96 standard stimuli with 24 stimuli being presented at each location, and bimodal audiovisual stimuli contained 10 target stimuli and 24 standard stimuli when the auditory stimuli were presented at one of the four locations (in total, 40 target stimuli and 96 standard stimuli at four locations). These stimuli were randomly presented in each block. Each block had a 3000 ms fixation period followed by the test stimulus. During the fixation period, there was a cross (+) on the screen. During the response period that followed the test stimulus, the screen was clear. The experiment then continued with the next trial occurring at the set ISI time (800–1200 ms), regardless of whether the subject had responded to the target stimulus. Throughout the experiment, the subjects were required to fix their eyes on a centrally presented fixation point on a screen and to attend to the visual stimuli containing the unimodal visual stimulus and the visual segment of the audiovisual stimuli, while ignoring all auditory stimuli (Figure 1). The participants were instructed to respond to visual target stimuli by pressing the left button of a computer mouse with their right hand as quickly and accurately as possible.

### Apparatus

Stimulus presentation was controlled by a personal computer running Presentation software (Neurobehavioral Systems, Albany, CA). An electroencephalographic (EEG) system (BrainAmp MR plus, Gilching, Germany) was used to record EEG signals through 32 electrodes mounted on an electrode cap (Easy-cap, Herrsching Breitbrunn, Germany). All signals were referenced to the left and right earlobes. Horizontal eye movements were measured by deriving the electrooculogram (EOG) from one electrode placed at the outer canthi of the left eye. Vertical eye movements and eye blinks were detected by deriving an EOG from an electrode placed approximately one centimeter below the subject’s left eye. The impedance was maintained below 5 kΩ. Raw signals were digitized using a sample frequency of 500 Hz with a 60 Hz notch filter, and all data were stored digitally for off-line analysis. The ERP analysis was carried out using Brain Vision Analyzer software (version 1.05, Brain Products GmbH, Munich, Bavaria, Germany).

### Data Analysis

#### Behavioral data

Hit rates and response times to target stimuli were computed separately for each stimulus type. Hit rates were the number of correct responses to target stimuli divided by the total number of target stimuli. Response time data were analyzed for correct responses. The data for incorrect responses to standard stimuli were submitted using false alarm rates. We then used a z-transformed ratio to compute sensitivity (d’) and criterion (c) [25]. Behavioral results for all measures (response times, hit rates, false alarm rates, d’ and response criterion) were analyzed using a repeated-measures analysis of variance with the stimulus modalities (visual stimuli and audiovisual stimuli) as subject factors, and the alpha level was set at *p*<0.05.

#### ERP analysis

The ERPs elicited by the standard stimuli were analyzed. The EEG and EOG signals were amplified and band-pass filtered with an analog filter of 0.01–100 Hz at a sampling rate of 500 Hz. EEG and EOG signals were divided into epochs from 100 ms before the stimulus onset to 600 ms after onset, and baseline corrections were made against −100–0 ms. Trials with vertical eye movements and eye blinks (vertical EOG amplitudes exceeding ±100 µV), horizontal eyeball movements (horizontal EOG amplitudes exceeding ±25 µV), or other artifacts (a voltage exceeding ±80 µV at any electrode location relative to baseline) were excluded from the analysis. These trials were subjected to automatic rejection. Responses associated with false alarms were also rejected from the analysis. The data were then averaged for each stimulus type following digital filtering using a band-pass filter of 0.01–30 Hz. The grand-averaged data were obtained across all participants for each stimulus type. Audiovisual integration was assessed by the difference wave [AV-(A+V)], obtained by subtracting the sum of the responses to the unimodal stimuli from the responses to the bimodal stimuli [26], [27]. The logic of this additive model is that the ERPs to bimodal (AV) stimuli are equal to the sum of the unimodal (A+V) responses plus the putative neural activities specifically related to the bimodal nature of the stimuli. Mean amplitudes were calculated for all electrodes at consecutive windows of 20 ms each between stimulus onset and 500 ms after presentation of the stimulus. The mean amplitude data were analyzed using repeated-measures analysis of variance with the within-subjects factors of modality (AV, A+V), time-window and electrodes. The Greenhouse-Geisser Epsilon correction was applied to adjust the degrees of freedom of the F ratios as necessary. All statistical analyses were carried out using SPSS version 16.0 software package (SPSS, Tokyo, Japan).

## Results

### Behavioral Results

The average reaction times are shown in Table 1. Analysis of the factor stimulus type confirmed that reaction times to unimodal visual and bimodal audiovisual stimuli (auditory stimuli from four locations) differed significantly [F (4, 52) = 8.079; *p* = 0.001]. In addition, post-hoc comparisons found that the reaction times to the bimodal audiovisual stimuli in which auditory stimuli were presented in the front (*p* = 0.01) and back (*p* = 0.031) of the participants were significantly faster than those to the unimodal visual stimuli. These results indicate that a synergistic effect occurred when the auditory stimuli were presented in the back space. However, the pairwise comparisons showed that no significant differences in reaction times were found between the bimodal audiovisual stimuli in which auditory stimuli were presented on the left (*p* = 0.333) or right (*p* = 0.066) of the participants and the unimodal visual stimuli.

**Table 1:** Behavioral mean data over all participants in the experiment.

Stimulus types	Reaction times (ms)	Hit rates (%)	False alarm rates (%)	Perceptual sensitivities (d’)	Response criterion (c)
V	529 (28.6)	78 (6.6)	0.74 (0.17)	3.62 (0.21)	0.71 (0.02)
A_F_V	498 (19.5)	86 (3.8)	1.07 (0.30)	3.82 (0.22)	0.72 (0.02)
A_B_V	500 (20.3)	85 (4.1)	0.99 (0.24)	3.68 (0.19)	0.78 (0.01)
A_L_V	515 (37.0)	81 (3.7)	0.83 (0.19)	3.61 (0.18)	0.74 (0.01)
A_R_V	514 (37.8)	79 (5.9)	1.11 (0.31)	3.39 (0.23)	0.75 (0.01)

SDs are given in parentheses. A_F_V, A_B_V, A_L_V, A_R_V: auditory stimuli of bimodal audiovisual stimuli were presented at the front, back, left, and right of the participant, respectively; V: unimodal visual stimuli.

The hit rates showed similar effects as the response times (Table 1). These effects were statistically expressed as a main effect of the within-subject factor stimulus type [F (4, 52) = 7.317; *p* = 0.002]. The pairwise comparisons showed that hit rates differed significantly between unimodal visual and bimodal audiovisual stimuli in which auditory stimuli were presented at the front (*p* = 0.02) or back (*p* = 0.027) of the participants (the responses to the bimodal audiovisual stimuli were faster), but no significant differences were found between unimodal visual stimuli and bimodal audiovisual stimuli in which auditory stimuli were presented on the left (*p* = 0.068) or right (*p* = 0.336) of the participants. However, regarding the false alarm rates, there was no significant effect of the factor stimulus type [F (4, 52) = 1.328, *p* = 0.28]. As Table 1 shows, the false alarm rates for stimulus type were low, particularly for unimodal visual stimuli, which produced the lowest false alarm rates. The hit and false alarm rates were then used to compute the signal detection measure d’. There was no significant effect of stimulus type on d’ [F (4, 52) = 0.852, *p* = 0.466] (Table 1). These results indicate that if the hit rate to one stimulus type was high, false alarm rate of this stimulus type was also high. Thus, no significant difference was found between stimulus types for d’. Response criterions are also shown in Table 1, which shows that a significant effect on response criterions were found for stimulus types [F (4, 52) = 6.143, *p* = 0.001]. Additionally, pairwise comparisons found that response criterions to the bimodal audiovisual stimuli in which auditory stimuli were presented at the front (*p* = 0.019) or back (*p* = 0.040) of the participants were lower than those to the unimodal visual stimuli.

### ERP Results

#### Event-related potentials: unimodal stimuli

**Figure 2:**
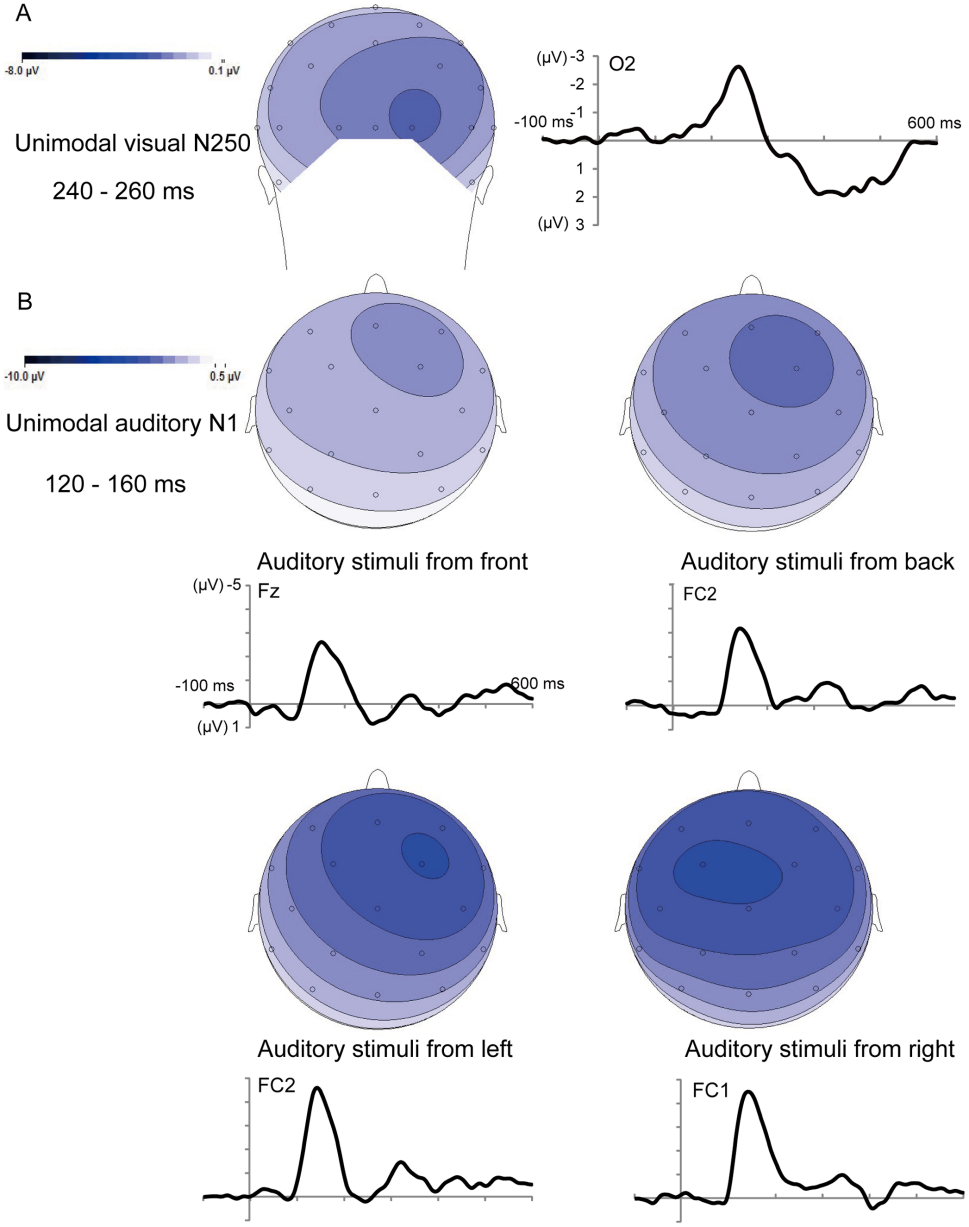
Waveforms and scalp topographies of unimodal stimuli. (A) Unimodal visual occipital N250 component. (B) Unimodal auditory N1 (auditory stimuli were presented at the front, back, left or right of the subjects). Note that the auditory N1 effect shows a clear contralateral enhancement (below).

Figure 2 shows the group-averaged ERPs to unimodal visual stimuli and unimodal auditory stimuli at a subset of selected electrode sites for which the potentials were recorded at greater amplitudes. The visual ERP showed a prominent negative wave peaking at approximately 250 ms after stimuli onset (N250 component) at occipital sites (peak: −2.63 µV at O2) (Figure 2A). For the auditory stimuli, which were presented at the front (0°), back (180°), left (270°) and right (90°) of the subjects, ERPs showed a prominent negative wave, and the negative N1 wave peaked at approximately 140 ms post-stimulus at the frontal sites (peak: −2.61 µV, −2.96 µV, −4.15 µV, −4.19 µV at Fz). Significantly, scalp topographies showed that the amplitude of the auditory N1 was greater over the hemisphere contralateral in response to stimulus presentation (Figure 2B, below).

#### Event-related potentials: audiovisual integration

**Figure 3:**
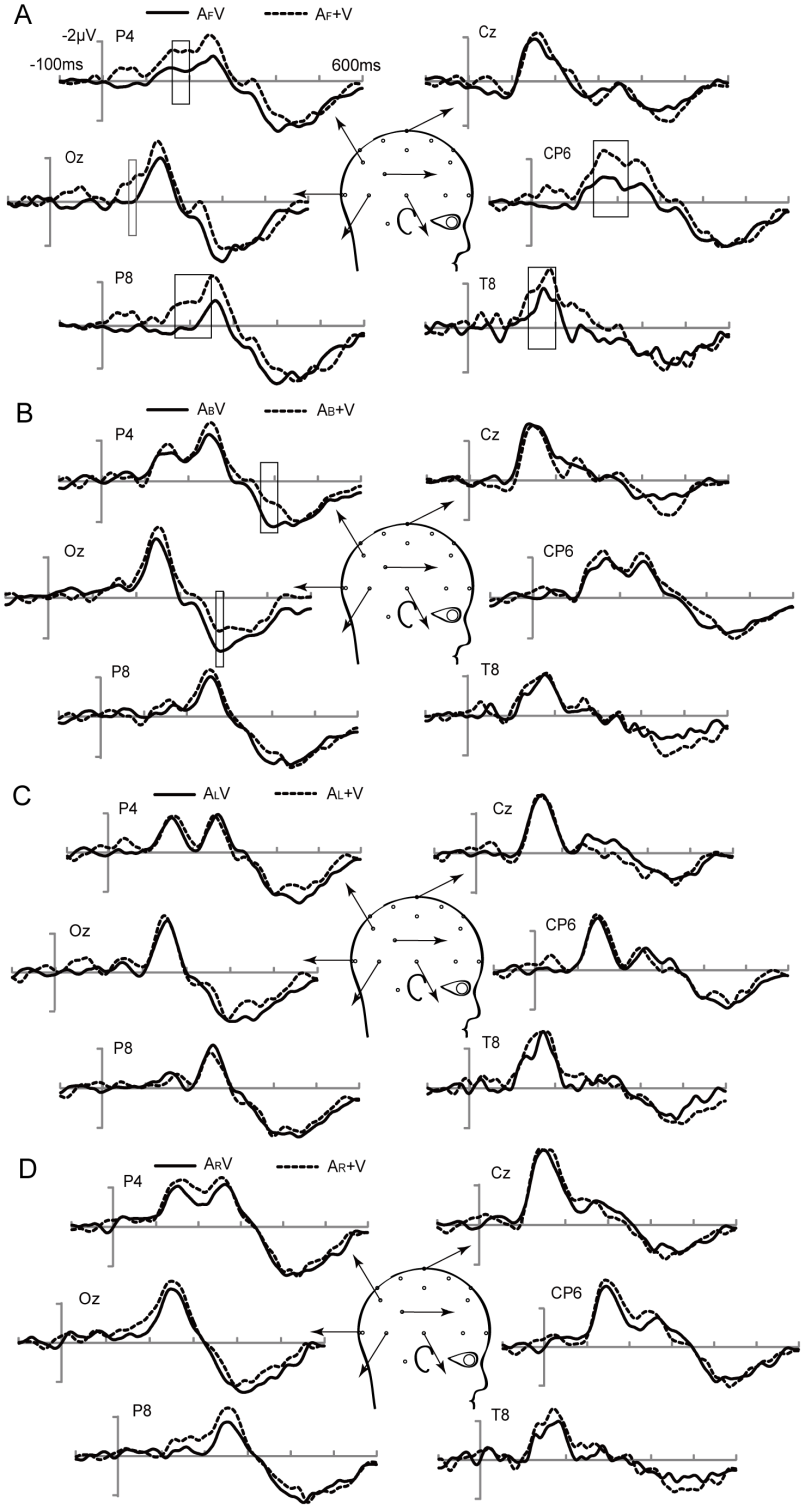
Grand-average event-related potentials elicited by audiovisual stimuli and (auditory plus visual). Event-related potentials of the sum of the unimodal stimuli (A+V) and bimodal (AV) stimuli at a subset of electrodes are shown from 100 ms before the stimulus to 600 ms after. (A) Auditory stimuli from the front. (B) Auditory stimuli from the back. (C) Auditory stimuli from the left. (D) Auditory stimuli from the right. The square areas indicate the time periods when the bimodal response significantly differs from the sum of the unimodal responses (p<0.01). (A_F_V, A_B_V, A_L_V, A_R_V: unimodal auditory stimuli of bimodal audiovisual stimuli were presented at the front, back, left and right of the subjects, respectively; A_F_, A_B_, A_L_, A_R_: unimodal auditory stimuli were presented at the front, back, left and right of the subjects, respectively).

Figure 3 shows the AV and (A+V) ERPs at several electrodes when auditory stimuli were presented at the front, back, left and right of the subjects. Several remarkable integration patterns were identified over the right temporal area and occipital area at approximately 160 to 200 ms when the auditory stimuli were presented in front of the subjects, and at the parietal and occipital areas at approximately 360 to 400 ms when the auditory stimuli were presented at the back of the subjects. These different integration patterns are analyzed in detail below. However, few or no significant integration patterns were found when the auditory stimuli were presented at the left or right of the subjects.

#### Integration in right temporal and occipital areas (160 to 200 ms)

**Figure 4:**
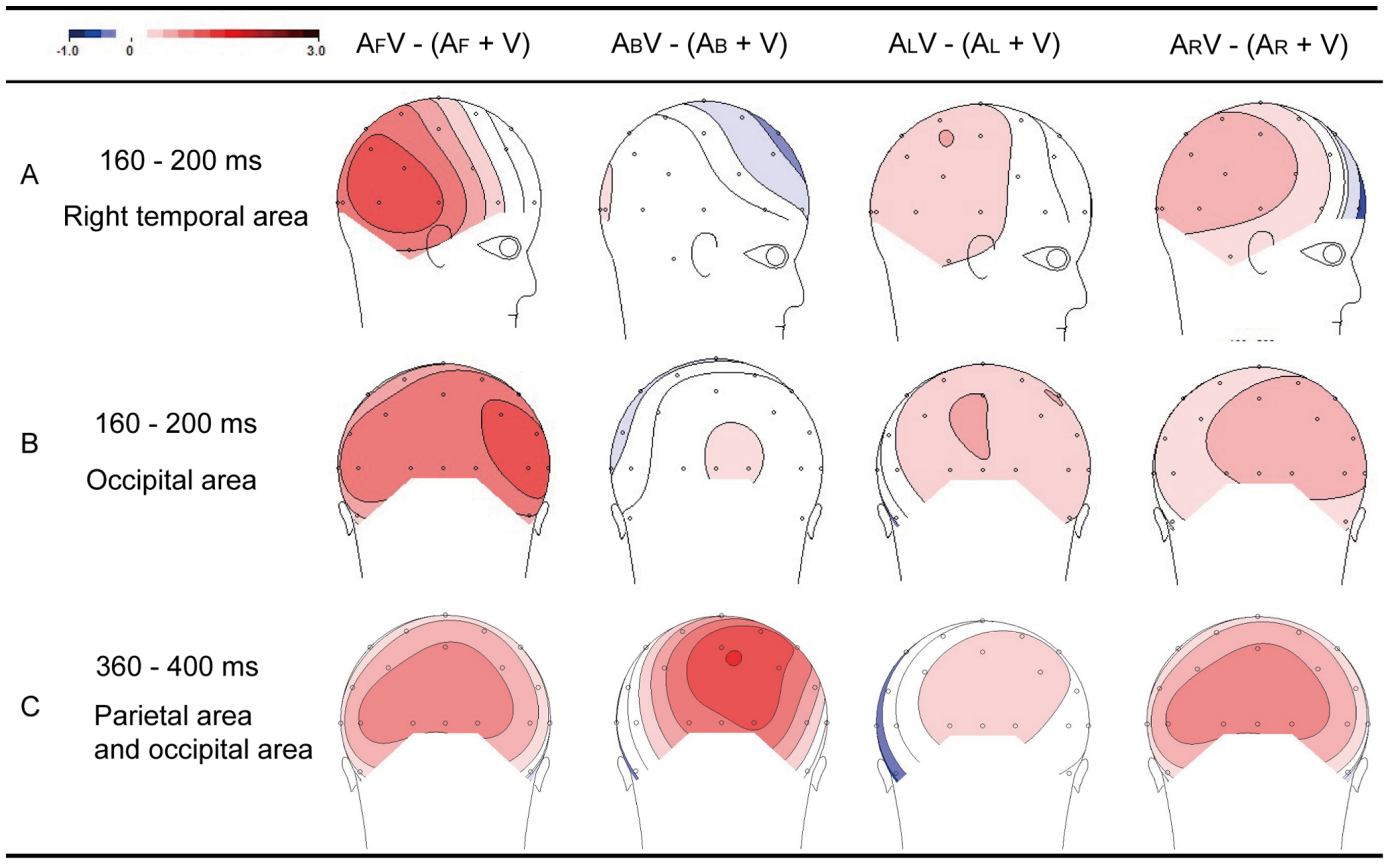
Topography of the different significant spatio-temporal patterns of integration. Audiovisual integration occurred (A) over the right temporal area at approximately 160–200 ms when the auditory stimuli were located in front of the participants; (B) over the right occipital area at approximately 160–200 ms when auditory stimuli were located in front of the participants; (C) over the right occipital area at approximately 360–400 ms when auditory stimuli were located in back of the participants. (A_F_, A_B_, A_L_, A_R_: unimodal auditory stimuli were presented in the front, back, left and right of the subjects, respectively).

When auditory stimuli were presented in front of the subjects, the observed effects were the most notable because the differences in amplitudes between AV and (A+V) were highly significant at the right temporal area and extended to the right occipital electrodes (P4, P8, CP6, Oz, O2) (Figure 3). Analysis of these amplitudes showed that the main effects of the modality [F (1, 13) = 13.23, *p*<0.01] and electrode [F (4, 52) = 10.33, *p*<0.01]. However, no significant interaction between modality and electrode were found [F (4, 52) = 0.713, *p* = 0.532]. The difference in amplitudes between AV and (A+V) were apparent at approximately 140 ms and reached a statistical significance of 0.01 between 160 and 200 ms at P4 [F (1, 13) = 13.01, *p* = 0.005] (mean amplitude, AV-(A+V): 0.95 µV), P8 [F (1, 13) = 15.32, *p* = 0.001] (mean amplitude: 1.16 µV) and CP6 [F (1, 13) = 19.09, *p* = 0.003] (mean amplitude: 1.10 µV). The occipital differences between AV and (A+V) were confirmed at Oz [F (1, 13) = 10.13, *p* = 0.010] (mean amplitude: 0.95 µV) and O2 [F (1, 13) = 6.52, *p* = 0.029] (mean amplitude: 0.91 µV). However, from approximately 160 to 200 ms latency, no significant differences between AV and (A+V) were found at the right temporal and occipital areas when auditory stimuli were presented to the back [F (1, 13) = 0.09, *p* = 0.762], left [F (1, 13) = 2.21, *p* = 1.169] and right [F (1, 13) = 3.46, *p* = 0.093] of the subjects (Figure 3). The topography of [AV-(A+V)] showing the effects of auditory stimuli from the four different locations is shown in Figure 4. The positive values in the [AV-(A+V)] differences at the right temporal and occipital areas were due to smaller amplitudes in the AV than in the (A+V) conditions. This result indicated that the amplitude of the response in auditory N1 was smaller for bimodal stimuli than for unimodal auditory stimuli.

#### Integration of parietal and occipital areas (360 to 400 ms)

When auditory stimuli were presented behind the subjects, at approximately 360 to 400 ms latency, significant differences in the amplitudes between the AV and (A+V) conditions [F (1, 13) = 8.32, *p*<0.05] were found at several parietal and occipital electrodes (Pz, P4, Oz, O2) (Figure 3). Statistical significance of *p*<0.01 was reached between 360 and 400 ms at P4 (mean amplitude, AV-(A+V): 1.02 µV) and also between 380 and 400 ms at Oz (mean amplitude: 0.98 µV) and O2 (mean amplitude: 1.09 µV). However, neither a significant effects of electrode type (Pz, P4, Oz, O2) nor significant interactions between electrode and modality were found.

In the same manner, no significant corresponding patterns were observed in this latency window when the auditory stimuli were presented at the other three locations (front [F (1, 13) = 3.41, *p* = 0.094], left [F (1, 13) = 0.54, *p* = 0.479] and right [F (1, 13) = 2.71, *p* = 0.131]). In addition, the topographies of the integration effect in this latency window to auditory stimuli at the back of the subjects appeared to be stronger than when the auditory stimuli originated from the other three locations (Figure 4).

## Discussion

The main focus of this study was to determine whether auditory stimuli in the horizontal plane affects audiovisual integration, particularly auditory stimuli presented at the back of subjects. Our behavioral and neural results demonstrated that integration occurred when the auditory stimuli were presented at the back and front of the participants. Two main event-related potential components related to audiovisual integration were found: first, auditory stimuli from the front location produced an ERP reaction over the right temporal area and right occipital area at approximately 160–200 ms; second, auditory stimuli from the back produced a reaction over the parietal and occipital areas at approximately 360–400 ms. No significant integration was found when auditory stimuli originated from the left or right spaces.

### Integration of Auditory Stimuli from the Front

A major integration was found in the 160–200 ms interval at the right temporal area that extended to the right occipital electrodes (Figure 3). Previous studies have reported audiovisual integration in which stimuli were presented in the frontal horizontal plane, and the observed reactions are closely analogous to this result [6], [7], [20], [28]. However, the latency for centrally presented stimuli at 145–160 ms occurred somewhat earlier than previously found [7]. This effect may have been due to attention, which has been shown to augment audiovisual integration processes, is that audiovisual integration under attended condition was greater than unattended condition [29], [30], [31]; additionally, and audiovisual integration depends on the stimuli being fully attended [32]. In the current study, only centrally presented visual stimuli were attended, unlike a study by Giard and Peronnet (1999), who investigated audiovisual integration under conditions of simultaneous attention to both modalities. In addition, a clear finding from our results is the strong predominance of the right hemisphere in audiovisual integration. This result is in agreement with findings from previous studies, which showed that neural activation was greater in the right hemisphere [7], [33], [34]. However, in one study in which subjects received auditory, visual, and audiovisual letters of the roman alphabet and were required to identify them regardless of stimulus modality, the left posterior superior temporal sulcus (STS) showed prominent audiovisual integration for letters [35]. Moreover, previous studies on speech stimuli have found that the left hemisphere is responsible for most integration functions [36], [37], [38]. These findings indicated that the left hemispheric is involved in the integration of certain types of information. Therefore, different characteristic stimulus types have significant influences on audiovisual integration between hemispheres.

### Integration of Auditory Stimuli from the Back

The most important, novel findings of the present study were the effects of auditory stimulation originating behind the participants (180°) on visual perception. In this portion of the study, behavioral facilitation was significant (*p*<0.05) (Table 1), and activity in the parietal and occipital areas was identified at approximately 360–400 ms after stimulus onset with audiovisual integration (Figure 4). These results were not in agreement with several previous studies [11], [19], [21]. In these studies, performance enhancement of simple detection tasks was reduced or absent with spatial separation of the stimuli. In these previous studies, visual and auditory stimuli were both presented in front of the participants and were separated by only 30° or 40°. Additionally, other previous studies have investigated the perception of auditory stimuli in the horizontal and sagittal planes. Findings from these studies showed that the perception of spatial auditory stimuli can be affected by the distance between the auditory stimuli from the two ears and demonstrated that the contribution of the near ear increases and, conversely, that of the far ear decreases [39], [40]. Thus, when the auditory stimuli were presented in the median vertical plane (front 0°; back 180°), the distance of the auditory signal from the front and back space to the two ears were equal, suggesting that the signal reached both ears simultaneously. Thus, the auditory event was temporally congruent with the visual signal that was presented in front of the participant. Temporal importance was also confirmed in several studies on multisensory interaction or integration, which found that the interaction or integration was greater when the visual and auditory stimuli occurred simultaneously [41], [42]. The results also demonstrated that spatial proximity of the two stimuli was critical for integration. Thus, the delayed response in the EEG signal to auditory signal from the back space might be due to spatial discordance between the visual and auditory stimuli. Furthermore, previous functional magnetic resonance imaging (fMRI) studies also investigated the perception and detection of auditory stimuli in the horizontal directions. The behavioral data from these studies showed that no significant difference was found between the detection of auditory stimuli from the front and back spaces. However, their fMRI results showed that the activity of the left posterior temporal gyri (pSTG) was greater for back than front auditory stimuli [43]. These results indicate that integration was less efficient from the back space compared to the front space, potentially because a greater number of neural resources are needed to perform the same task. Nevertheless, when visual and auditory stimuli were simultaneously presented in front of the subjects, a spatially consistent sound might rapidly facilitate attention and perception to visual stimuli [20]. Therefore, in this study, audiovisual integration began later in the back space than in the front space according to the EEG signal, which was consistent with the behavioral results on “back” effects.

In a similar manner, Zampini et al. (2007) studied auditory and somatosensory stimuli pairings that were presented to either the same or different positions in the front and/or back of the participants. The results from the study showed that responses were significantly more rapid to bimodal auditory and somatosensory stimuli than to a unimodal modality, regardless of whether the stimuli were presented from the front or back spaces [44]. The findings indicated that auditory stimuli from the back could also improve the perception of the somatosensory stimuli to the same level as that achieved by auditory stimuli from the front space. These results, together with those of Jones et al. (2006) and the present study, confirm that the facilitation of behavioral responses occurred even though modalities were presented in the front and back locations. Thus, these results suggest that new neural brain activities might be activated when modalities are presented in the back and that integration could occur and does not completely depend on the spatial position of the stimuli. However, further electrophysiological studies are needed to confirm and elucidate these neural mechanisms. More specifically, studies on stimuli presented in the back space are often neglected in multisensory research.

### Integration of Auditory Stimuli from the Left and Right Spaces

Our results showed that the contralateral auditory cortex was activated when unimodal auditory stimuli were presented in the left or right locations; this finding is consistent with previous studies [45]. However, no significant integration was found when visual stimuli were centrally presented and auditory stimuli were presented in the left (90°) or right (90°) location. Recently, behavioral studies have found that the facilitative effects of peripherally paired visual-auditory stimuli were less than those presented centrally (0°) and have even found that no facilitation was produced by the right 90° location [14]. In this study, it was confirmed by ERP analysis that no significant integration occurred when auditory stimuli were presented from the lateral locations. A possible reason for this result is that the two ears, which are in lateral symmetry on the head have inter-aural differences, such as auditory stimuli originating from the right (90°) side and thus different amounts of times were needed to inputs from both the left and right ears [46]. Thus, under this condition, the auditory signal is both temporally and spatially incongruent with the visual signal. Moreover, previous studies have found that the timing of auditory and visual stimuli has effects on integration [9], [47] and also confirmed that integration was greater when the auditory stimuli were presented in close temporal and spatial proximity with the visual stimuli [41], [42]. Thus, it had been suggested that temporal and spatial processing of the two signals were pivotal in determining whether integration would occur. These findings were consistent with our results. When a visual stimulus was presented at the front and an auditory stimulus was presented at the front or back, integration or interaction occurred; however, this integration was delayed when the auditory signal was presented in the back space. However, when the auditory information was presented at the sides (and the visual signal was presented at the front), integration was not observed. Further electrophysiological studies are needed to elucidate these neural mechanisms.

### Conclusions

This study shows the influences of auditory stimuli presented in the horizontal plane at the front (0°, the fixation point), right (90°), back (180°), and left (270°) of the participant on audiovisual integration processing. The data clearly showed that audiovisual integration occurred over the right temporal area and right occipital area at approximately 160–200 ms latency when the auditory stimuli were located in front of the participant. However, no significant integration was found when auditory stimuli were presented in the right and left spaces. More specifically, when the auditory stimuli were presented from the back, audiovisual integration was observed over the parietal and occipital areas at approximately 360–400 ms. We believe that our findings, particularly regarding auditory stimuli originating from the back of participants, are likely to be a useful reference for further studies that investigate the integration mechanism when different modality stimuli are presented at different spatial positions.
